# Characterization of circulating RSV strains among subjects in the OUTSMART-RSV surveillance program during the 2016-17 winter viral season in the United States

**DOI:** 10.1371/journal.pone.0200319

**Published:** 2018-07-24

**Authors:** Alexey Ruzin, Susan T. Pastula, Elizabeth Levin-Sparenberg, Xiaohui Jiang, Jon Fryzek, Andrey Tovchigrechko, Bin Lu, Yanping Qi, Hui Liu, Hong Jin, Li Yu, Judith Hackett, Tonya Villafana, Mark T. Esser

**Affiliations:** 1 AstraZeneca/MedImmune, Gaithersburg, Maryland, United States of America; 2 Epidstat Institute, Ann Arbor, Michigan, United States of America; 3 AstraZeneca/MedImmune, Mountain View, California, United States of America; Louisiana State University System, UNITED STATES

## Abstract

**Background:**

Respiratory syncytial virus (RSV) is an established cause of serious lower respiratory disease in infants, elderly and high-risk populations. The OUTSMART surveillance program aims to characterize patient populations and currently circulating RSV strains, and monitor temporal and geographic evolution of RSV F and G proteins in the U.S.

**Methods:**

The OUTSMART 2016–17 study collected RSV-positive samples from 25 RSVAlert^®^ laboratories from 4 U.S. regions and Puerto Rico during November 2016 through March 2017. Frequencies of A and B subtypes and genotypes were determined for several demographic and geographic variables. To gauge the representativeness of the OUTSMART patients, results were compared to discharge data from the NEDS and NIS databases.

**Results:**

A total of 1,041 RSV-positive samples with associated demographic data were obtained and the RSV F gene and second variable region of the G gene were sequenced. The majority of samples (76.0%) came from children under 2 years old: <1 year (48.4%), 1–2 years (27.6%). The OUTSMART patient sample was similar to NEDS and NIS for age, gender, and geographic location. Both OUTSMART and national RSV cases peaked in January. Of OUTSMART samples, 45.3% were subtype A, 53.7% were subtype B and 1.0% were mixed A and B. The percentage of RSV B cases increased with increasing age. Hospitalization (length of hospital stay, LOS, >24 hrs) occurred in 29.0% of patients of which 52.0% had RSV B. Outpatients (LOS <24 hrs) were 64.4% of total of which 73.3% were diagnosed in the ER and discharged, while only 6% were diagnosed in other outpatient settings.

**Conclusions:**

The OUTSMART 2016–17 study was representative of the U.S. RSV experience. Geographic and temporal information from the RSV surveillance program will be used to establish a molecular baseline of RSV F and G sequence variability and to help inform development of novel agents for RSV prophylaxis and treatment.

## Introduction

Respiratory syncytial virus (RSV) is an established cause of serious lower respiratory disease, particularly among children [1]. RSV typically exhibits distinct seasonality in temperate regions, with onset beginning in late fall or early winter, and ending in late spring [1].

In 2015 there were an estimated 33 million RSV infections globally in children under 5 years old, resulting in about 3 million hospitalizations and 60,000 deaths [2]. In the U.S., approximately 2.1 million children under age 5 require medical care for RSV each year, with 3% hospitalized, 25% treated in emergency departments (ED), and 73% seen in pediatric practices [3]. Reinfection is common throughout life, although symptoms in adults and older children are often milder or absent [4].

Recent studies reported that among viral respiratory admissions of young children, RSV hospitalizations are 6–14 times higher than for influenza [5, 6]. In the U.S., annual costs for RSV in children under 5 are estimated to be $400 million for RSV hospitalizations, $258 million for ambulatory medical care and more than $300 million for direct hospital charges [7, 8].

RSV is a non-segmented, single strand negative virus comprised of 11 proteins including 3 surface proteins (F, G, SH), of which F and G are the most important as they elicit both neutralizing and non-neutralizing antibodies. RSV has two major subtypes, A and B, based on antigenic and genetic variation in the G attachment protein [9]. The F fusion protein is highly conserved with 90% sequence identity between the subgroups [10], elicits broadly neutralizing antibodies, and is the target of the licensed mAb, palivizumab [11]. The F protein is also the target of a more potent neutralizing mAb, MEDI8897, with half-life extension technology that is currently being evaluated in pre-term infants [12, 13]. In contrast, the heavily glycosylated G attachment protein is highly variable, differing by 53% at the amino acid level between A and B subtypes [10].

A number of studies show RSV A and B can cocirculate during a single epidemic and temporal and geographic clustering of RSV genotypes can occur [14]. The evolution of RSV genotypes through accumulated changes in amino acids of the G protein are likely due to immune pressure from neutralizing antibodies elicited following infection [15, 16]. RSV is sub-classified into 13 RSV A genotypes and 20 RSV B genotypes based on the second hyper-variable region of the G gene [17, 18]. Currently, predominate RSV B genotypes are derived from the Buenos Aires strain, first identified in 1999, which has a 60 base pair duplication in the second hyper-variable region of the G gene [19]. The predominant RSV A genotypes are derived from Ontario 1 (ON1), first described in 2006, which has a 72 base pair duplication in the G protein [20]. Numerous studies compared the severity of RSV A and B infections in hospitalized children with inconsistent results as to which subtype is more likely to cause severe infections [14]. These conflicting reports suggest temporal and geographic differences may be important in understanding the association of RSV genotype and disease and that monitoring the molecular evolution of RSV would be useful in assisting the development of anti-RSV drugs and prophylactic approaches.

The first experimental RSV vaccine was tested in the 1960s and not only failed to protect against RSV in clinical trials, but led to enhanced disease following subsequent RSV infection such that 80% of infants who received the vaccine were hospitalized and two died [21, 22]. A successful passive immunization approach with immunoglobulin was developed over 25 years later with the approval of Respigam^™^ in 1996 [23] followed by the approval of a monoclonal antibody (mAb), palivizumab (Synagis^™^) in 1998 [24]. Currently, Palivizumab is the only prophylactic agent approved by the FDA for prevention of RSV in high-risk infants and children [25]. Although rare, Palivizumab resistant viruses have been identified in the clinical setting [26]. Several novel vaccines and mAbs are in development to prevent RSV disease in infants and the elderly [27–29]. To assist with medical decision making regarding current RSV prophylaxis and to help inform the development of new agents, the RSVAlert system was developed [30]. RSVAlert currently tracks RSV testing and results from approximately 480 hospital laboratories across the U.S. (https://rsvalert.com). The Observational United States Targeted Surveillance of Monoclonal Antibody Resistance and Testing of RSV (OUTSMART-RSV) program was developed to collect samples and associated case information and to provide F and G sequence data from a subset of laboratories participating in RSVAlert. OUTSMART was piloted in 2015–2016 and allows more complete characterization of currently circulating strains, including their temporal and geographic evolution in the U.S., and further characterization of the RSV patient population.

## Materials and methods

### Study design

**Fig 1:**
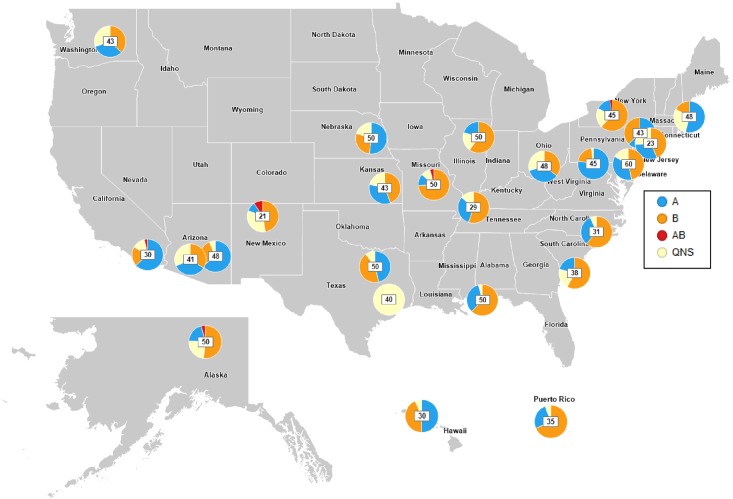
Map of participating OUTSMART laboratories during the 2016–2017 season. Pie-charts represent proportions of RSV A (blue), RSV B (orange), RSV A+B (red) and QNS (yellow) samples per lab. Numbers within the pie charts represent the total number of samples per lab.

The OUTSMART 2016–17 study collected and analyzed a series of RSV-positive samples and associated anonymized, demographic data from a subset of hospital-based laboratories participating in RSVAlert and included 25 laboratories from 4 U.S. regions and Puerto Rico during November 2016 to March 2017 (Fig 1). Participating laboratories were selected and recruited based on their geographic location to represent all US regions including: West (including Alaska and Hawaii), Midwest, South, Northeast and Puerto Rico. The number of sites per each region were selected to provide approximately equal representation by region. Historical reporting of >50 RSV-positive samples per season to RSVAlert system was also taken into consideration during the site selection. Additionally, RSVAlert provided numbers of RSV-positive tests and total RSV tests conducted per month for each of the participating laboratories (S1 Data).

### Sample collection and analysis

Participating laboratories were instructed to provide a single RSV-positive respiratory sample (in UTM or VTM) per patient for a maximum of ten samples each month during the five months of the study period, resulting in a maximum total of 50 samples from each laboratory throughout the study period. The sites were instructed to provide the first 10 RSV-positive samples collected from the beginning of each month. The variability in the number of samples received from each site is primarily attributed to RSV-positive sample availability at that site for each month (some sites received less than 10 samples in a given month) and also by the compliance of each site to the study protocol. One laboratory, provided forty purified RSV RNA samples. In addition to samples, information collected included lab location (U.S. region and division, state, county, city, zip), date of sample collection, sample number, de-identified patient information such as age, gender, and length of hospital stay (LOS)(S2 Data). In this study, inpatients and outpatients were defined as those with LOS of either >24 hours or <24 hours in the hospital, respectively.

### Sequencing and bioinformatic analyses

Next generation sequencing (NGS) using the MiSeq (Illumina) was conducted on the PCR-amplified second hypervariable region of the G gene and the full-length F gene. Samples that did not generate at least 1,000 mapped reads with at least 4-fold depth of coverage of both F and G genes were marked as QNS and were excluded from the analysis. Contigs were constructed from the de-multiplexed MiSeq reads using Geneious software (Version 10.0.9, Biomatters Inc. Newark, NJ). A multiple sequence alignment (MSA) was built from the translated G protein sequences using MAFFT [31], and pairwise dissimilarity matrix was computed using Bishop–Friday substitution model [32]. To reduce the effects of PCR and sequencing artifacts, sequences were clustered at 97% similarity cutoff. A single representative sequence was picked within each cluster to build a neighbor-joining phylogenetic tree [33]. Detailed sequencing and bioinformatic methods are available in S1 Text.

### Comparison to national databases

To assess the representativeness of the OUTSMART patient sample with that of the U.S., results were compared to discharge data from the November 2013-March 2014 Nationwide Emergency Department Sample (NEDS) [34] and the National Inpatient Sample (NIS) [35]. The NIS is a nationally representative sample of hospital inpatient stays and the NEDS is a nationally representative sample of hospital-based ED visits. Both were developed by the Healthcare Cost and Utilization Project (HCUP) and sponsored by the Agency for Healthcare Research and Quality (AHRQ). The NEDS contains data from approximately 30 million all-payer ED visits annually, and when weighted, represents approximately 135 million ED visits. The NIS contains records from more than 7 million all-payer hospital stays annually and represents more than 35 million hospitalizations when weighted. Both databases contain multiple diagnostic codes for each hospitalization or ED visit, based on the *International Classification of Diseases*, *Ninth Revision*, *Clinical Modification* (ICD-9-CM; hereafter, described as ICD-9). During the study period, there were 3 RSV-specific ICD-9 codes: 480.1: Pneumonia due to RSV; 466.11: Bronchiolitis due to RSV; and 079.6: RSV. Our analysis included all hospitalizations and ED visits with at least one of the three RSV-specific ICD-9 codes listed in any diagnostic position in the patient record. Frequencies of ED visits and hospitalizations were calculated by age group, gender, U.S. region, and month based on the weighted estimate of total number of hospitalizations or ED visits due to RSV during the study period.

### Statistical methods

Frequencies of A and B subtypes were calculated by age group, gender, LOS, and U.S. region. The frequency of samples per month for each lab was also determined as was the percent positive among all tests conducted for each month, and for each month by RSV subtype. The percent of RSV B between age groups was compared using logistic regression with a Bonferroni correction to adjust for multiple comparisons. Chi-square tests were used to compare the age distributions in OUTSMART with the national samples. All data management and statistical analyses for this study were carried out using SAS version 9.4 (SAS Institute Inc., Cary, NC, USA), with procedures that incorporated NIS- and NEDS-provided weights to account for the structure of the sample survey data.

## Results

The twenty-five laboratories that participated in OUTSMART throughout the U.S. West (including Alaska and Hawaii), Midwest, South, Northeast regions and Puerto Rico (Fig 1) reported a total of 9,758 RSV-positive tests (10.7%) out of 90,840 tests conducted during November 2016—March 2017. Of the 25 participating laboratories, 16 submitted less than 50 samples (range: 23–48), 6 submitted 50 samples, and 1 laboratory submitted more than 50 samples (n = 60), resulting in a subset of 1,041 RSV positive samples with associated demographic data that were submitted to the OUTSMART surveillance program for F and G sequencing analyses to characterize variability of the F and G antigens and to determine the temporal and geographic distributions of RSV A and B genotypes. Of the 1,041 RSV positive samples, 836 samples (80.3%) yielded specific PCR amplified fragment of sufficient quantity and quality suitable for sequencing analysis. The remaining samples (205 samples; 19.7%) were marked as QNS (quantity/quality non-sufficient) and were not used in sequencing analysis as they failed to produce enough material suitable for sequencing. Thus, all samples with sufficient quantity and quality of DNA were sequenced and analyzed.

**Fig 2:**
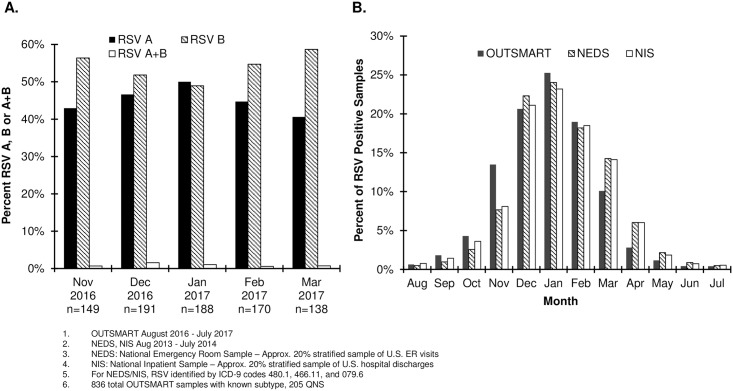
Temporal distributions of RSV positive tests. (A) OUTSMART 2016–17 RSV positive tests by RSV subtype. (B) All RSV positive tests in OUTSMART—participating laboratories, and RSV in NEDS and NIS.

The monthly positive samples were reported by RSV subtype and the temporal distribution of both A and B subtypes was generally similar (Fig 2A). The number of positive samples for subtype B and subtype A peaked in December 2016 and January 2017, respectively. To determine if the seasonality pattern identified in the OUTSMART study was generalizable to the U.S. RSV experience, OUTSMART data was compared with NEDS and NIS databases. The proportion of positive RSV tests out of all RSV tests conducted by OUTSMART participating laboratories had an approximately normal distribution which peaked in January and was similar to that of the NEDS and NIS databases (Fig 2B).

OUTSMART subject demographics were also similar to NEDS and NIS. The largest disease burden was in those <1 year (OUTSMART: 48.4%, NEDS 59.7%, NIS 57.8%), followed by the 1–2 year age group (OUTSMART: 27.6%, NEDS 28.2%, NIS 22.3%) (Table 1). The databases were also similar by gender (Percent male—OUTSMART: 53.1%, NEDS 53.8%, NIS 54.6%) (Table 1), and region. The largest proportions of cases occurred in the South (OUTSMART: 27.2%, NEDS: 34.4%, NIS: 37.8%), though the national samples had larger proportions of samples from the South than OUTSMART (Table 1). Subtype B was more frequent in the Midwest and South regions. The Northeast and West had almost equal distributions of A and B subtypes (Fig 1 and S1 Table).

**Table 1:** Comparison of OUTSMART November 2016—March 2017 RSV positive tests with RSV in NEDS and NIS November 2013-March 2014 by age, gender and region.

		OUTSMART		NEDS^a^^,^^c^		NIS^b^^,^^c^	
		N	%	N	%	N	%
**Age**	**<1 year**	504	48.4%	66,982	59.7%	39,130	57.8%
	**1–2 year**	287	27.6%	31,628	28.2%	15,070	22.3%
	**3–5 year**	74	7.1%	6,293	5.6%	3,750	5.5%
	**6–59 year**	90	8.6%	2,958	2.6%	4,195	6.2%
	**60+ year**	86	8.3%	4,352	3.9%	5,545	8.2%
	**All**	**1,041**	**100.0**%	**112,213**	**100.0**%	**67,690**	**100.0**%
**Gender**	**Male**	553	53.1%	60,449	53.8%	37,030	54.6%
	**Female**	488	46.9%	51,841	46.2%	30,830	45.4%
	**All**	**1,041**	**100.0**%	**112,290**	**100.0**%	**67,860**	**100.0**%
**Region**	**Mid-West**	241	23.2%	34,258	30.5%	15,800	23.3%
	**North East**	219	21.0%	19,509	17.4%	13,005	19.2%
	**South**	283	27.2%	38,611	34.4%	25,640	37.8%
	**West**	263	25.3%	19,912	17.7%	13,415	19.8%
	**PR**	35	3.4%	-	-	-	-
	**Total**	**1,041**	**100.0**%	**112,290**	**100.0**%	**67,860**	**100.0**%

^a^NEDS: National Emergency Room Sample—Approx. 20% stratified sample of U.S. emergency room visits

^b^NIS: National Inpatient Sample—Approx. 20% stratified sample of U.S. hospital discharges

^c^RSV identified by ICD-9 codes 480.1, 466.11, 079.6 in NEDS and NIS

**Fig 3:**
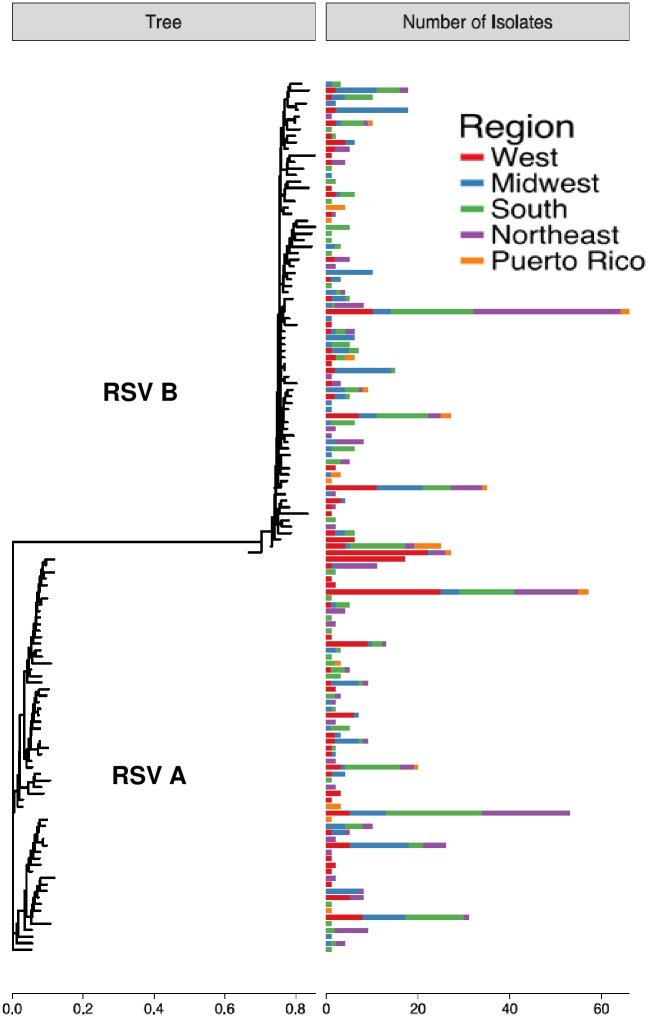
RSV A and B genotypes of 2016–17 OUTSMART samples by geographic region. The phylogenetic tree in the left panel was built using the representative G protein sequences from 97%-identity clusters, with the horizontal scale under the tree showing branch lengths derived from the dissimilarity metric. The corresponding bars in the right panel represent the number of samples in each cluster, with horizontal scale under the bar plot showing sample counts.

There were 387 subtype A and 457 subtype B viral sequences determined from the five different geographic regions. These sequences were assigned to genotypes based on the sequence of the second hyper-variable region of G gene. All RSV A samples belonged to the Ontario 1 (ON1) genotype [20] and all RSV B samples belonged to the Buenos Aires 9 (BA9) genotype [19], except one which belonged to the Buenos Aires 10 (BA10) genotype. In addition, we combined RSV A and RSV B sequences into distinct sub-genotypes or clusters based upon a 97% identity in the G second hyper-variable region (61 clusters for RSV A and 73 clusters for RSV B) and mapped them to different geographic regions (Fig 3). This analysis revealed that the 5 most frequent RSV A clusters comprised 48% of the 387 RSV A samples and the 5 most frequent RSV B clusters comprised 39% of the 457 RSV B samples. There were no obvious differences in geographic distribution of these strains in the West, Midwest, South and Northeast suggesting they were broadly distributed across the different U.S. regions.

The age distributions of OUTSMART samples were compared separately for inpatient and ER admissions with the two different national databases. OUTSMART inpatients included fewer infants < 1 year old than NIS (46.7% vs. 57.8%) and more older patients over age 60 (13.9% vs. 8.2%) (Table 2). The distribution of ER patients by age was similar in the OUTSMART and NEDS databases (Table 2).

**Table 2:** OUTSMART November 2016—March 2017 RSV positive inpatient and Emergency Room cases compared with NIS and NEDS RSV positive cases during November 2013-March 2014 by age group.

**Age**	**OUTSMART Inpatient**^a^		**NIS**^b^^,^^d^	
	**N**	**%**	**N**	**%**
**<1 year**	141	46.7%	39,130	57.8%
**1–2 year**	73	24.2%	15,070	22.3%
**3–5 year**	20	6.6%	3,750	5.5%
**6–59 year**	26	8.6%	4,195	6.2%
**60+ year**	42	13.9%	5,545	8.2%
**Total**	**302**	**100.0**%	**67,690**	**100.0**%
**Age**	**OUTSMART Emergency Room/department**		**NEDS**^c^^,^^d^	
	**N**	**%**	**N**	**%**
**<1 year**	305	55.6%	66,982	59.7%
**1–2 year**	176	32.1%	31,628	28.2%
**3–5 year**	32	5.8%	6,293	5.6%
**6–59 year**	24	4.4%	2,958	2.6%
**60+ year**	12	2.2%	4,352	3.9%
**Total**	**549**	**100.0**%	**112,213**	**100.0**%

^a.^ OUTSMART Inpatient: Length of stay>24 hr

^b.^ NIS: National Inpatient Sample—Approx. 20% stratified sample of U.S. hospital discharges

^c.^ NEDS: National Emergency Room Sample—Approx. 20% stratified sample of U.S. ER visits

^d.^ RSV identified by ICD-9 codes 480.1, 466.11, 079.6 in NIS

**Fig 4:**
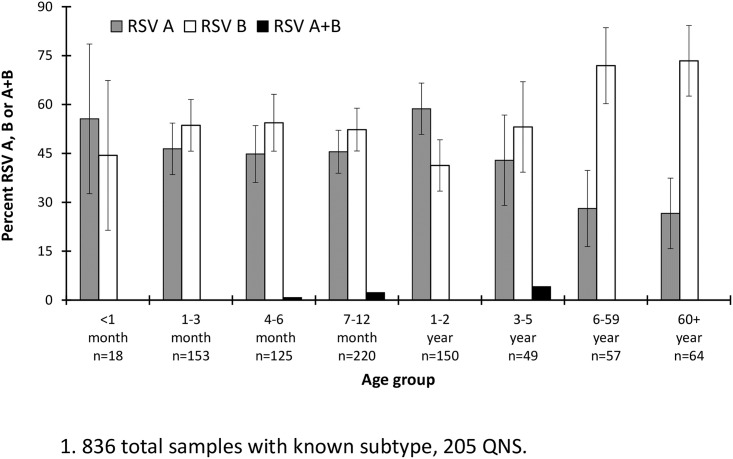
OUTSMART 2016–17 percent RSV-positive tests by age and subtype. Error bars represent 95% confidence intervals.

Of the OUTSMART samples with determined RSV subtype (n = 836; 80.3%), 45.3% were subtype A, 53.7% were subtype B, and 1% had both A and B subtypes (Fig 4 and S2 Table). Most samples (76.0%) came from children ≤2 years of age: <1 year (48.4%) and 1–2 years (27.6%) (Table 1). RSV B was more frequent in all ages with the exception of <1 month and 1–2 year old children, in which RSV A was more common (Fig 3). The highest proportion of RSV B cases (73.4%) was observed in subjects ages 60+ followed by the 6-59-year-old group (71.9%) (Fig 4 and S2 Table).

Severity of illness associated with RSV subtype, gender, or age was explored by categorizing RSV hospitalizations into LOS <24 hours and >24 hours. Hospitalizations >24 hours occurred among 29.0% of patients. Young children, ≤ 2 years of age, were the most frequently seen with RSV as both inpatients (214/302, 70.9%) and outpatients (546/670, 81.5%). (Table 3). LOS was stratified by referring department (Table 4) and 73.3% (n = 491) of RSV subjects with <24 hour LOS had samples collected from the ED and were discharged whereas only 6% of the samples came from an outpatient setting such as a doctor’s office. In contrast, only 19.2% of inpatient RSV cases were diagnosed in the ER with a significant number of samples coming from the pediatric intensive care unit and ICU. However, no information was provided for 55.3% of inpatient RSV cases (Table 4). These data suggest that a significant proportion of RSV disease in both the young and the old is managed in the emergency room without admitting the subject into the hospital.

**Table 3:** OUTSMART 2016–17 RSV-positive tests by LOS, age and subtype.

LOS	Age	RSV subtype							All	
		A		B		A+B		QNS		
		N	%	N	%	N	%	N	N	%
**<24 hr**^a^	**≤2 year**	227	47.3%	248	51.7%	5	1.0%	66	546	52.4%
	**3–59 year**	24	33.8%	46	64.8%	1	1.4%	31	102	9.8%
	**≥60 year**	3	20.0%	12	80.0%	0	0%	7	22	2.1%
	**Total**	**254**	**44.9**%	**306**	**54.1**%	**6**	**1.1**%	**104**	**670**	**64.4**%
**>24 hr**^a^	**≤2 year**	86	52.8%	76	46.6%	1	0.6%	51	214	20.6%
	**3–59 year**	10	40.0%	15	60.0%	0	0%	21	46	4.4%
	**≥60 year**	9	27.3%	24	72.7%	0	0%	9	42	4.0%
	**Total**	**105**	**47.5**%	**115**	**52.0**%	**1**	**0.5**%	**81**	**302**	**29.0**%
**NA**	**≤2 year**	12	52.2%	11	47.8%	0	0%	8	31	3.0%
	**3–59 year**	3	30.0%	6	60.0%	1	10.0%	6	16	1.5%
	**≥60 year**	5	31.3%	11	68.8%	0	0%	6	22	2.1%
	**Total**	**20**	**40.8**%	**28**	**57.1**%	**1**	**2.0**%	**20**	**69**	**6.6**%
**Total**		**379**	**45.3**%	**449**	**53.7**%	**8**	**1.0**%	**205**	**1,041**^b^	**100.0**%

^a.^ <24 hours defined as outpatient, >24 hours defined as inpatient

^b.^ 836 total samples with known subtype, 205 QNS

**Table 4:** OUTSMART 2016–17 RSV-positive tests by LOS, referring department and subtype.

		RSV Subtype (Sequencing Results)								
		A		B		AB		QNS	All	
LOS	Referring department	N	%	N	%	N	%	N	N	% of LOS category
**<24 hr**^a^	**Emergency room/department (ER/ED)**	207	47.9%	220	50.9%	5	1.2%	59	491	73.3%
	**Pediatric Intensive Care Unit (PICU)**	0	0%	1	100.0%	0	0%	0	1	0.1%
	**Outpatient Facility**	13	37.1%	21	60.0%	1	2.9%	5	40	6.0%
	**Other**	34	34.7%	64	65.3%	0	0%	40	138	20.6%
**Total <24 hr**									**670**	**64.4**%
**>24 hr**^a^	**Emergency room/department (ER/ED**	26	54.2%	22	45.8%	0	0%	10	58	19.2%
	**Pediatric Intensive Care Unit (PICU)**	21	67.7%	10	32.3%	0	0%	16	47	15.6%
	**ICU (Other than PICU)**	1	12.5%	7	87.5%	0	0%	6	14	4.6%
	**Pediatric Ward**	4	25.0%	12	75.0%	0	0%	0	16	5.3%
	**Other**	53	44.9%	64	54.2%	1	0.8%	49	167	55.3%
**Total >24 hr**									**302**	**29.0**%
	**Other**	20	40.8%	28	57.1%	1	2.0%	20	69	100.0%
**Total Other**									**69**	**6.6**%

^a.^ <24 hours defined as outpatient, >24 hours defined as inpatient

## Discussion

The OUTSMART RSV surveillance program characterizes circulating RSV strains and monitors their temporal and geographic evolution in the U.S. to help inform the development of anti-RSV mAbs and vaccines. RSV surveillance is also important in providing timely information to physicians for the administration of Palivizumab to eligible high-risk infants [36, 37]. The OUTSMART program was generally representative of the U.S. RSV infection experience in terms of age, gender distribution and seasonality compared to national data (Fig 2B). The OUTSMART RSV program is designed to run for several years to monitor temporal and regional differences in predominant subtype [38, 39], specifically in the southeastern U.S. where the RSV season typically begins earlier and lasts longer [40, 41] than in other areas of the country. RSV surveillance is additionally conducted by the Centers for Disease Control and Prevention (NREVSS) [42], the international Respiratory Syncytial Virus Network (ReSVinet) [43] and the European Influenza Surveillance Network (EISN) [44]. While all show differences in onset and length of RSV seasons depending on regional setting, none provide molecular typing of strains or characterize them temporally or geographically. This study, which characterized both G and F genotypes, along with surveillance data from these other networks can help inform timing of administration of a mAb or vaccine in clinical trials and provide a baseline for molecular heterogeneity of viruses currently in circulation prior to testing and licensure of an RSV mAb or vaccine [36, 37].

Compared to the national databases for inpatient and emergency admissions, OUTSMART-participating laboratories differed in their age distributions of annual positive RSV cases (Chi-square p-value <0.001 and 0.010, respectively) (Table 2). This may have been due to patient sampling or a shift in age-specific infection rates since the national data were from a different season compared to OUTSMART. As the OUTSMART program progresses, accumulation of results from additional seasons will allow for a more accurate comparison to national trends.

There were limitations to this study design, which was intended to cover all 4 regions and 9 divisions of the country defined by the U.S. census. Some areas were not as well represented as others such as the upper Midwest and West. When comparing data to national trends, NIS and NEDS estimates are based on ICD9 codes and not laboratory-confirmed diagnoses, so comparability to the OUTSMART data is limited. OUTSMART could not be compared to the national sample from the same season because the 2016–17 national data was not yet available. Lastly, the use of hospital-based laboratory data on RSV infections markedly underestimates the full burden of RSV disease in the U.S. However, because these cases are laboratory-verified, the data are useful in providing additional information on hospital and ER burden of RSV disease.

Hospitalizations are often used as the key measure of severity and to estimate the economic impact of RSV infection. This significantly underestimates the true burden of RSV disease by not evaluating cases, which may include severe cases, which are medically managed in the ER without hospital admission or in physician offices as outpatients. In 2003, Leader and Kohlhase examined several national databases and found that between 1997–2000, there were 718,000 ER visits for lower respiratory infections in infants <1 year, with a cost of $202 million. Only 29% of these patients were admitted [45]. An additional challenge to accurate estimation of total RSV burden is that routine RSV testing is rarely performed in outpatient settings and is not recommended by the American Academy of Pediatrics (AAP) [46].

One of the strengths of the OUTSMART study is that it was designed to be an ongoing surveillance program with widespread participation and laboratory-confirmed diagnoses of RSV. A database of trends in infection rates will be built to inform drug and vaccine development programs. Information from OUTSMART will also be used to establish a baseline of RSV F and G sequences as a reference for future epidemiology studies and clinical trials. A separate report will describe the conservation of the F protein and the susceptibility of different RSV isolates to neutralization by a novel mAb MEDI8897, currently being developed to prevent medically attended lower respiratory tract infections due to RT-PCR confirmed RSV in all infants [13, 47]. In addition, an ex-U.S. RSV surveillance program entitled INFORM-RSV has been launched in collaboration with ReSViNET (www.resvinet.org) to collect RSV samples from Europe, South America, South Africa, Australia and Japan.

An interesting observation in this study was that there was a significantly larger proportion of RSV B detected in the 6–59 (p = 0.001) and 60+ (p<0.001) age groups as compared to the 1–2 year old age group (Fig 4). The difference in RSV A and B prevalence in the elderly versus the very young may be the result of pre-existing immunity to RSV A gained from previous infections. It will be interesting to see if the prevalence of A and B in different age groups changes over time and whether that correlates with changes in the F and G genes.

Nearly one-third of RSV-positive cases identified in the OUTSMART program were hospitalized for greater than 24 hours. Hospitalization rates for RSV positive patients published by Radin et al. [48] were similar to those estimated using data from OUTSMART. Radin et al. reported that 28% of all RSV cases were hospitalized in their study of three separate U.S. populations. They also found that 71% of identified RSV cases were under age 4 [48], similar to the infection rate of 76% found amongst OUTSMART patients of ≤ 2 years of age.

Most RSV cases in OUTSMART were diagnosed in the ER and did not result in the subject being admitted to the hospital for more than 24 hours. Over 70% of RSV cases with <24 hours LOS were diagnosed in the ED, and only 6% in doctor’s offices or clinics likely due to primarily hospital-based case collection. Most ER diagnoses were in children less than 2 years old. Parents may be choosing the costlier treatment setting of the ER for their children over waiting for a pediatrician appointment due to perceived urgency of symptoms, or lack of private health insurance or primary care provider. In total, 64.4% of RSV cases that spent less than 24 hours in the hospital were seen in the ER. This is an important finding from the OUTSMART study in that much of the burden of RSV disease does not appear to lie in hospitalizations, but in the ER. OUTSMART has identified a signal for future research to gain more clarity of the full burden of RSV disease in all healthcare settings.

An additional explanation for the large proportion of cases diagnosed in the ED compared with other outpatient settings such as a physician’s office, is that very little testing for RSV is conducted in these settings as it does not alter treatment decisions [3, 7, 49]. A better understanding of the burden of disease and related costs in the outpatient setting is necessary to better inform the design of clinical studies and the future impact of novel interventions.

Despite the inability to completely capture all circulating RSV cases due to lack of uniform diagnostic testing in all healthcare settings, OUTSMART provides a reasonable description of verified RSV diagnoses based on current medical practice. Future RSV surveillance and epidemiology studies will need to address the burden of disease in all settings, including outpatient clinics and the ER.

## Supporting information

S1 Data Total number of tests conducted and total number of positive tests at OUTSMART participating labs. (XLSX)

S2 Data 2016–2017 OUTSMART surveillance data. (XLSX)

S1 Table OUTSMART 2016–17 RSV-positive tests by region and age and subtype. (DOCX)

S2 Table OUTSMART 2016–17 RSV-positive tests by gender, age and subtype. (DOCX)

S1 Text Supplementary methods. (DOCX)

## Abbreviations

ED: Emergency Department
ER: Emergency Room
HCUP: Healthcare Cost and Utilization Project
ICD9-CM: International Classification of Diseases, Ninth Revision, Clinical Modification
LOS: length of stay
mAb: monoclonal antibody
NA: Not Available
NEDS: National Emergency Department Sample
NIS: National Inpatient Sample
OUTSMART: Observational United States Targeted Surveillance of Monoclonal Antibody Resistance and Testing
PCR: Polymerase Chain Reaction
QNS: Quality/Quantity Not Sufficient
RNA: Ribonucleic Acid
RSV: Respiratory Syncytial Virus
UTM: Universal Transport Medium
VTM: Viral Transport Medium
