# Supplementary material for: Characterization of circulating RSV strains among subjects in the OUTSMART-RSV surveillance program during the 2016-17 winter viral season in the United States
Source: PLoS One. 2018 Jul 24;13(7):e0200319. doi: 10.1371/journal.pone.0200319 (PMC6057637; doi:10.1371/journal.pone.0200319)
Supplement: S1 Table — (DOCX) [file pone.0200319.s003.docx]

**Table S1**: OUTSMART 2016-17 RSV-positive tests by Region and Age and Subtype

| Region | Age | RSV subtype (Sequencing Results) | | | | | | | All | |
| --- | --- | --- | --- | --- | --- | --- | --- | --- | --- | --- |
|  |  | A | | B | | AB | | QNS |  |  |
|  |  | N | % | N | % | N | % | N | N | % |
| Midwest^a^ | <=2 year | 55 | 42.0% | 74 | 56.5% | 2 | 1.5% | 28 | 159 | 15.3% |
|  | 3-59 year | 12 | 30.0% | 28 | 70.0% | 0 | 0% | 16 | 56 | 5.4% |
|  | 60+ year | 7 | 31.8% | 15 | 68.2% | 0 | 0% | 4 | 26 | 2.5% |
|  | **Total** | **74** | **38.3**% | **117** | **60.6**% | **2** | **1.0**% | **48** | **241** | **23.2**% |
| Northeast^b^ | <=2 year | 80 | 58.4% | 57 | 41.6% | 0 | 0% | 20 | 157 | 15.1% |
|  | 3-59 year | 6 | 22.2% | 20 | 74.1% | 1 | 3.7% | 10 | 37 | 3.6% |
|  | 60+ year | 3 | 18.8% | 13 | 81.3% | 0 | 0% | 9 | 25 | 2.4% |
|  | **Total** | **89** | **49.4**% | **90** | **50.0**% | **1** | **0.6**% | **39** | **219** | **21.0**% |
| South^c^ | <=2 year | 98 | 46.0% | 115 | 54.0% | 0 | 0% | 48 | 261 | 25.1% |
|  | 3-59 year | 4 | 57.1% | 3 | 42.9% | 0 | 0% | 14 | 21 | 2.0% |
|  | 60+ year | 0 | 0% | 1 | 100.0% | 0 | 0% | 0 | 1 | 0.1% |
|  | **Total** | **102** | **46.2**% | **119** | **53.8**% | **0** | **0**% | **62** | **283** | **27.2**% |
| West^d^ | <=2 year | 83 | 54.6% | 65 | 42.8% | 4 | 2.6% | 27 | 179 | 17.2% |
|  | 3-59 year | 15 | 46.9% | 16 | 50.0% | 1 | 3.1% | 18 | 50 | 4.8% |
|  | 60+ year | 7 | 28.0% | 18 | 72.0% | 0 | 0% | 9 | 34 | 3.3% |
|  | **Total** | **105** | **50.2**% | **99** | **47.4**% | **5** | **2.4**% | **54** | **263** | **25.3**% |
| Puerto Rico | <=2 year | 9 | 27.3% | 24 | 72.7% | 0 | 0% | 2 | 35 | 3.4% |
|  | **Total** | **9** | **27.3**% | **24** | **72.7**% | **0** | **0**% | **2** | **35** | **3.4**% |
| **Total** | | **379** | **45.3**% | **449** | **53.7**% | **8** | **1.0**% | **205** | **1041** | **100.0**% |

^a^ Includes test locations from children’s hospitals.

1. Mid-West: IN, KS, MO, NE, OH
2. Northeast: CT, MA, NY, PA
3. South: FL, GA, MD, NC, TN, TX
4. West: AK, AZ, CA, HI, NM, WA
