# Supplementary material for: Characterization of circulating RSV strains among subjects in the OUTSMART-RSV surveillance program during the 2016-17 winter viral season in the United States
Source: PLoS One. 2018 Jul 24;13(7):e0200319. doi: 10.1371/journal.pone.0200319 (PMC6057637; doi:10.1371/journal.pone.0200319)
Supplement: S2 Table — (DOCX) [file pone.0200319.s004.docx]

**Table S2**: OUTSMART 2016-17 RSV-positive tests by gender, age and subtype

| Gender | Age | RSV subtype (Sequencing Results) | | | | | | | All | |
| --- | --- | --- | --- | --- | --- | --- | --- | --- | --- | --- |
|  |  | A | | B | | AB | | QNS |  |  |
|  |  | N | % | N | % | N | % | N | N | % |
| Male | <=2 year | 172 | 47.6% | 184 | 51.0% | 5 | 1.4% | 73 | 434 | 41.7% |
|  | 3-59 year | 20 | 39.2% | 30 | 58.8% | 1 | 2.0% | 27 | 78 | 7.5% |
|  | 60+ year | 10 | 33.3% | 20 | 66.7% | 0 | 0% | 11 | 41 | 3.9% |
|  | **Total** | **202** | **45.7**% | **234** | **52.9**% | **6** | **1.4**% | **111** | **553** | **53.1**% |
| Female | <=2 year | 153 | 50.2% | 151 | 49.5% | 1 | 0.3% | 52 | 357 | 34.3% |
|  | 3-59 year | 17 | 30.9% | 37 | 67.3% | 1 | 1.8% | 31 | 86 | 8.3% |
|  | 60+ year | 7 | 20.6% | 27 | 79.4% | 0 | 0% | 11 | 45 | 4.3% |
|  | **Total** | **177** | **44.9**% | **215** | **54.6**% | **2** | **0.5**% | **94** | **488** | **46.9**% |
| **Total** | | **379** | **45.3**% | **449** | **53.7**% | **8** | **1.0**% | **205** | **1041^a^** | **100.0**% |

1. 836 total samples with known subtype, 205 QNS
