# Supplementary material for: Characterization of circulating RSV strains among subjects in the OUTSMART-RSV surveillance program during the 2016-17 winter viral season in the United States
Source: PLoS One. 2018 Jul 24;13(7):e0200319. doi: 10.1371/journal.pone.0200319 (PMC6057637; doi:10.1371/journal.pone.0200319)
Supplement: S1 Text — (DOCX) [file pone.0200319.s005.docx]

# S1 Text. Supplementary Methods

## Sequencing and bioinformatic analyses

RSV positive respiratory samples were stored and shipped in either Universal Transport Media (UTM) or Viral Transport Media (VTM). Viral RNA from the respiratory samples was extracted and purified using the Nuclisens easyMAG® instruments (bioMerieux) according to the manufacturer’s instructions. RT-PCR was then performed on the purified RNA using the SuperScript III One-Step RT-PCR System (Invitrogen, CA) with forward primer, RSV_F5109-5129Y (5’ AGTGTTCAAYTTYGTWCCYTG 3’) and reverse primer RSV_R7654-7634 (5’ YTACCATTCAAGCAATGACCTC 3’), which were designed to amplify the RSV A and B genomes harboring the 2nd hypervariable region of the G gene and the full length F gene in a single 2.5kb fragment. Next Generation Sequencing (NGS) was then conducted on the amplified RT-PCR product. Contigs were constructed from the de-multiplexed MiSeq reads using Geneious software (Version 10.0.9, Biomatters Inc. Newark, NJ). Curated assemblies were validated and annotated by visual inspection and quality control before sequence analysis.

The sequences were examined for quality and coverage, and assigned an RSV subtype based on the alignment to RSV A and RSV B reference sequences. To make a RSV A and B co-infection call, the ratio of minimum coverage (depth) of the less dominant strain to dominant strain was set at ≥5%. The translated amino acid sequence quality was checked in the Remote Analysis of Drug Activity and Resistance Database (RADAR, MedImmune) system. Only sequences containing the second hyper variable region of G protein and complete sequence of full length F protein coding region were analyzed further. Samples that did not generate at least 1000 mapped reads with at least 4-fold depth of coverage of both F and G genes were marked as QNS and were excluded from the analysis.

Genotypes were assigned based on a best match in a nucleotide BLAST alignment of the second hypervariable region of the G gene against a database of reference sequences with known genotypes (1). The assigned genotypes were confirmed by inspecting the clustering of sequences observed in a phylogenetic tree built from a join set of all study sequences and the reference database sequences.

A multiple sequence alignment (MSA) was built from the translated G protein sequences using MAFFT (2), and pairwise dissimilarity matrix was computed using Bishop-Friday substitution model implemented in R package DECIPHER (3). For sequence QC purposes, the presence of outliers in the distance matrix was checked using our custom method built on top of Local Outlier Factor (4) approach implemented in R package Rlof (<https://cran.r-project.org/package=Rlof>). No outliers were detected. In order to reduce overplotting on the phylogenetic tree figures and reduce the effects of PCR and sequencing artefacts, sequences were clustered by complete linkage, and split into clusters at 97% similarity cutoff. A single medoid representative (exemplar) sequence was picked within each cluster, and used to build a neighbor-joining phylogenetic tree with R package *phangorn* (5). The count of all sequences within each cluster was retained to use as multiplier in plots showing regional sequence distribution. Tree figures were generated with R package ggtree (6). The automated analysis and graphical reporting pipeline was implemented with our R-based MGSAT (<https://github.com/andreyto/mgsat>) and Python-based MICGENT (<https://github.com/andreyto/micgent>) packages. The analysis code and reference data specific to this project are available at (<https://github.com/andreyto/rsv_epi_2017_suppl>).

# References

1. Tabatabai J, Prifert C, Pfeil J, Grulich-Henn J, Schnitzler P. Novel respiratory syncytial virus (RSV) genotype ON1 predominates in Germany during winter season 2012-13. PLoS One. 2014;9(10):e109191.

2. Katoh K, Standley DM. MAFFT multiple sequence alignment software version 7: improvements in performance and usability. Mol Biol Evol. 2013;30(4):772-80.

3. Wright ES. Using DECIPHER v2.0 to Analyze Big Biological Sequence Data in R. The R Journal. 2016;8(1):352-9.

4. Breunig MM, Kriegel H-P, Ng RT, Sander J, editors. LOF: identifying density-based local outliers. ACM sigmod record; 2000: ACM.

5. Schliep KP. phangorn: phylogenetic analysis in R. Bioinformatics. 2011;27(4):592-3.

6. Yu G, Smith DK, Zhu H, Guan Y, Lam TTY. ggtree: an R package for visualization and annotation of phylogenetic trees with their covariates and other associated data. Methods in Ecology and Evolution. 2017;8(1):28-36.
